# A Web-Based Mental Health Platform for Individuals Seeking Specialized Mental Health Care Services: Multicenter Pragmatic Randomized Controlled Trial

**DOI:** 10.2196/10838

**Published:** 2019-06-04

**Authors:** Jennifer M Hensel, James Shaw, Noah M Ivers, Laura Desveaux, Simone N Vigod, Ashley Cohen, Nike Onabajo, Payal Agarwal, Geetha Mukerji, Rebecca Yang, Megan Nguyen, Zachary Bouck, Ivy Wong, Lianne Jeffs, Trevor Jamieson, R Sacha Bhatia

**Affiliations:** 1 Women's College Institute for Health Systems Solutions and Virtual Care Toronto, ON Canada; 2 Department of Psychiatry University of Toronto Toronto, ON Canada; 3 Department of Psychiatry University of Manitoba Winnipeg, MB Canada; 4 Women's College Research Institute Toronto, ON Canada; 5 Department of Family and Community Medicine University of Toronto Toronto, ON Canada; 6 Institute for Health Policy, Management and Evaluation University of Toronto Toronto, ON Canada; 7 Li Ka Shing Knowledge Institute St Michael's Hospital Toronto, ON Canada; 8 Department of Medicine University of Toronto Toronto, ON Canada

**Keywords:** internet, mental health, anxiety, depression

## Abstract

**Background:**

Web-based self-directed mental health applications are rapidly emerging to address health service gaps and unmet needs for information and support.

**Objective:**

The aim of this study was to determine if a multicomponent, moderated Web-based mental health application could benefit individuals with mental health symptoms severe enough to warrant specialized mental health care.

**Methods:**

A multicenter, pragmatic randomized controlled trial was conducted across several outpatient mental health programs affiliated with 3 hospital programs in Ontario, Canada. Individuals referred to or receiving treatment, aged 16 years or older, with access to the internet and an email address, and having the ability to navigate a Web-based mental health application were eligible. A total of 812 participants were randomized 2:1 to receive immediate (immediate treatment group, ITG) or delayed (delayed treatment group, DTG) access for 3 months to the Big White Wall (BWW), a multicomponent Web-based mental health intervention based in the United Kingdom and New Zealand. The primary outcome was the total score on the Recovery Assessment Scale, revised (RAS-r) which measures mental health recovery. Secondary outcomes were total scores on the Patient Health Questionnaire-9 item (PHQ-9), the Generalized Anxiety Disorder Questionnaire-7 item (GAD-7), the EuroQOL 5-dimension quality of life questionnaire (EQ-5D-5L), and the Community Integration Questionnaire. An exploratory analysis examined the association between actual BWW use (categorized into quartiles) and outcomes among study completers.

**Results:**

Intervention participants achieved small, statistically significant increases in adjusted RAS-r score (4.97 points, 95% CI 2.90 to 7.05), and decreases in PHQ-9 score (−1.83 points, 95% CI −2.85 to −0.82) and GAD-7 score (−1.55 points, 95% CI −2.42 to −0.70). Follow-up was achieved for 55% (446/812) at 3 months, 48% (260/542) of ITG participants and 69% (186/270) of DTG participants. Only 58% (312/542) of ITG participants logged on more than once. Some higher BWW user groups had significantly greater improvements in PHQ-9 and GAD-7 relative to the lowest use group.

**Conclusions:**

The Web-based application may be beneficial; however, many participants did not engage in an ongoing way. This has implications for patient selection and engagement as well as delivery and funding structures for similar Web-based interventions.

**Trial Registration:**

ClinicalTrials.gov NCT02896894; https://clinicaltrials.gov/ct2/show/NCT02896894 (Archived by WebCite at http://www.webcitation.org/78LIpnuRO)

## Introduction

Mental illness is prevalent, with estimates suggesting that upward of 1 billion people worldwide could be affected at a given point in time [1]. In addition, mental and substance use disorders are emerging as a leading cause of disability, accounting for nearly 10% of global disability-adjusted life years [1]. Access to and use of appropriate and timely mental health services and specialists, however, continues to be a challenge because of limited resources and individual-level factors surrounding treatment seeking [2-4]. E-mental health applications can potentially help to address some of these gaps [5]. A number of Web-based interventions including smartphone apps and Web-based treatment programs for common mental disorders have demonstrated small-to-moderate treatment effect sizes for symptom reduction [6-8], although these interventions are commonly recommended as standalone or preventative treatment options for those with milder symptoms, where benefits have been most apparent. Engagement with self-directed, Web-based interventions has been cited as a challenge owing to a range of user and intervention design factors, with multicomponent interventions potentially enhancing engagement through more user choice, added interactivity, and customization [9,10]. Moreover, the general advancement and adoption of virtual care is often in the absence of rigorous evaluation and adequate planning for sustainability and spread [11,12].

Investments in digital health worldwide have included a substantial emphasis on digital and virtual intervention to promote health [13], and Canada is no exception [14]. As a part of a series of demonstration projects being implemented by leading stakeholders in digital health and telemedicine in Ontario, Canada’s most populous province, the Big White Wall (BWW) was selected as a solution with the potential to be adopted for mental health. The BWW [15] is a multicomponent moderated internet-based intervention with peer support that provides anonymity. At the time this trial was conducted, there had been no previous randomized trial evaluating the BWW.

The target goal for the Ontario demonstration projects was chronic disease management and high-needs patient populations, so the sponsors intentionally selected specialized mental health treatment settings for the intervention. This study sought to determine the utility of the BWW as a solution in a Canadian setting, specifically for individuals with mental health symptomatology severe enough to warrant a need for specialized mental health care. We investigated the effectiveness of 3 months of access to the BWW for mental health recovery, as well as symptoms of depression and anxiety, quality of life, and integration with one’s community, relative to a usual care control group who received delayed access to the intervention after the study period. We hypothesized that the BWW would increase mental health recovery across a variety of mental health–related needs and conditions **.**

## Methods

### Trial Design

This study was a multicenter, parallel-arm, pragmatic randomized controlled trial. Participants seeking services at specialized mental health and addiction programs at the participating sites were randomized 2:1 to receive immediate access to the BWW for 3 months (immediate treatment group, ITG) or delayed access after a 3-month control period (delayed treatment group, DTG). The trial protocol has been previously published and is available open access [16]. The trial was sponsored by Ontario Telemedicine Network and Canada Health Infoway, both government-funded organizations. Sponsors specified the recruitment target and study settings, but had no involvement in the study design, procedures, data collection, or analysis.

### Ethics

This study received ethical approval from the research ethics boards at all participating sites. All participants gave informed consent before taking part in the study.

### Changes to Trial Design After Commencement

In response to automated follow-up surveys going to email *junk boxes*, personalized emails were sent with the survey link embedded. The frequency of contact to remind participants to complete follow-up surveys was increased, and surveys were completed over the phone when possible. To meet sponsor recruitment targets, recruitment was extended early in the study to individuals attending clinics rather than the initial approach targeting those on waitlists or being discharged. Given a large amount of referral between programs, early on in recruitment, it became apparent that participants were commonly on waitlists for specialized clinics while receiving active treatment in other recruitment settings, so this change had little impact on the overall composition of the study sample.

### Recruitment of Participants and Baseline Assessment

Participants were recruited from outpatient mental health programs affiliated with 3 hospitals in Ontario: (1) a public psychiatric hospital in a medium-sized city with satellite treatment sites in smaller urban centers; (2) a large community hospital located in another medium-sized city with satellite addictions programs; and (3) a large ambulatory academic hospital located centrally in a large metropolitan area. The aim of the study was to determine the utility of the BWW as a broad reaching multicomponent Web-based intervention that crosses all mental health and addiction-related needs. The BWW contains guided educational and course content applicable to a wide range of mental health needs, and the peer support component offers various opportunities to engage in discussion relevant to one’s personal needs. Thus, we recruited from a range of outpatient programs which included: adult mood and anxiety psychiatry programs, a substance use program, an emergency department, an urgent care clinic, youth mood and anxiety programs, mood and anxiety psychotherapy programs, trauma therapy programs, and a borderline personality disorder program. Program coordinators, clinicians, and administrators at each site reviewed referral waitlists and clinic rosters to identify potential participants and refer them to the study coordinator, who reached out by telephone or met the individual at the clinic. Eligible participants were aged 16 years or older, had access to the internet and an email address, were able to read English, and willing and able to access and use a Web-based mental health intervention. There were no exclusion criteria but referring clinicians were asked to consider if person would be able to participate appropriately in Web-based peer interactions. There were no imposed restrictions on the use of concomitant care, including accessing other Web-based interventions that may be like the one under investigation.

All participants provided informed consent and baseline variables either in person or by phone with a study team member. Baseline assessment included sociodemographic data and all outcome measures. Participants were also asked about baseline belief in the treatment credibility and outcome expectancy. Participants were asked to rate their agreement with the author-generated question, “Self-help tools including web-based services and books are helpful for people with mental health problems.” Item 4 from the Credibility and Expectancy Questionnaire [17] was adapted for this study, “By the end of the [BWW access period], how much improvement in your symptoms do you think will occur?” Participants were asked to rate their response from 0% to 100% with options available in 10% increments. This single item has been shown to correlate strongly with mental health treatment outcomes such as psychotherapy [18]. Participants were given the option to complete additional symptom, function, and service utilization measures over the phone or by computerized survey. Secure computerized assessments were sent electronically to each participant’s email address. All data were entered into a REDCap database [19]. Trial recruitment took place between July 2016 and January 2017.

### Participant Safety

The team at each study site monitored adverse events. A data safety and monitoring board consisting of 3 experts external to the research team reviewed the trial data at 3 and 6 months after recruitment started, specifically examining change in item 9 (suicidal ideation) scores on the Patient Health Questionnaire-9 item (PHQ-9), as well as all serious adverse events reported by the participating sites throughout the trial.

### Intervention—The Big White Wall

**Figure 1:**
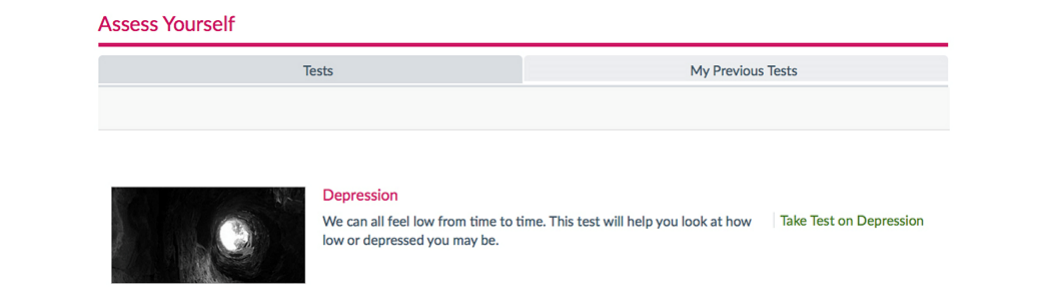
The Big White Wall offers self assessments across a range of mental health concerns including depression, anxiety, and substance use.

**Figure 2:**
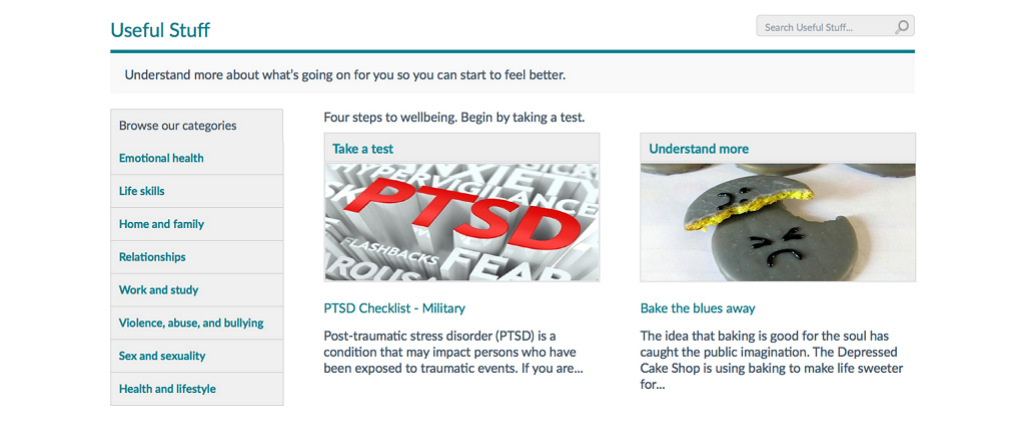
The Useful Stuff pages provide information on mental health conditions and interventions.

**Figure 3:**
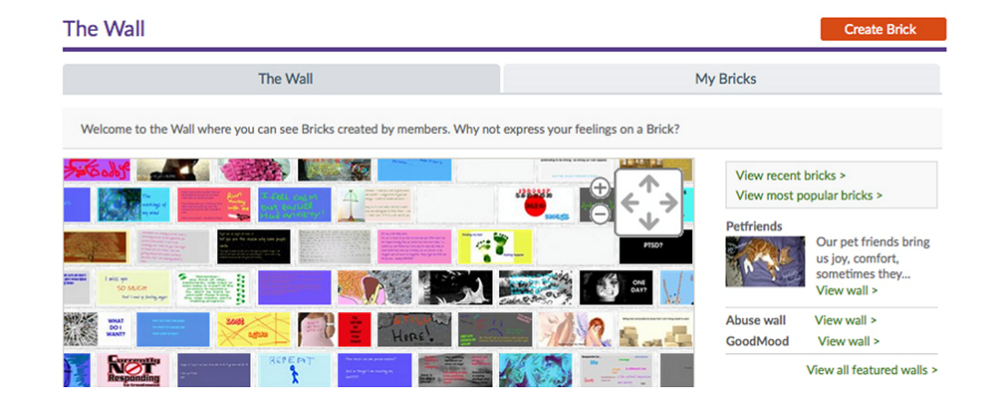
The Wall is a space for users to post self-expression statements through the creation of artistic bricks. Users can also comment on each other’s bricks.

**Figure 4:**
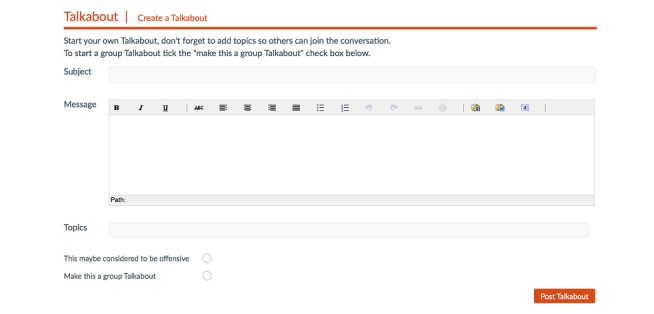
Moderated personal and group Talkabouts allow users to converse with wall guides and peers regarding their mental health concerns and experiences.

The BWW is a multicomponent, self-directed, and moderated Web-based intervention founded in 2007 and hosted in the United Kingdom. Access to the BWW was free for study participants for 3 months (ITG during the 3-month trial period and DTG after the initial 3-month trial period). The developers advocate that the BWW can improve mental health symptoms through increasing social engagement, normalizing experiences, educating, and equipping with skills to manage difficulties. All participants maintain anonymity on the site through a unique nonidentifiable user alias and use is participant dependent [15]. The BWW is monitored 24/7 by *Wall Guides* who are trained mental health professionals employed by the BWW and based in the United Kingdom and New Zealand. These individuals constantly review user activity and posts to ensure the content is appropriate and sensitive to all users. They will engage with users through instant communication and in the case of identified risk, the Wall Guides can identify the location of the user and direct them to use local crisis services. Contact information for local crisis services was provided on the unique landing page created for the Ontario users. The BWW components include (1) educational material, (2) guided support courses based on principles of cognitive behavioral therapy and behavior change, and (3) text communication posts as either 1:1 with another member or a Wall Guide or open to discussion groups composed of any members of the peer community. The educational materials and guided support courses cover a wide range of mental health related topics including grief, depression, anxiety, smoking cessation, substance use, trauma, among others. Users can also post and comment on *bricks*—creative self-expressions whereby the user designs a brick which they can place in the digital *wall*. See Figures 1-4 for screenshots of the BWW components. The BWW reaches out to users through their registration email if there has been a prolonged period of inactivity, encouraging users to log on. In this study, all participants received an email alias that linked to their personal email and a unique prescription for a BWW account. A follow-up call was made by a study team member 3 days after sending the prescription and again after 2 weeks, if the account had not been activated. Technical support was available from the research team, the BWW, and the Ontario Telemedicine Network.

### Follow-Up

Follow-up data were collected between October 2016 and April 2017. All follow-up data were collected by self-report via electronic surveys through REDCap or collected by phone or in person by a study team member and subsequently input into the REDCap database.

Participants received an automated survey link by email 1 week before 3 months postrandomization with a follow-up personalized email containing the survey link. In these emails, DTG participants were reminded that they would receive access to the BWW once the survey was completed, although all participants were ultimately given access regardless of survey completion. At 3 months and 3 months plus 1 week, reminder phone calls were made. Surveys were closed 2 weeks after the 3-month time point. Study team members collecting the outcome assessments were blinded to group allocation. Both baseline and 3-month postrandomization surveys were completed via the Web-based survey in 95% of cases, with the remainder by phone or in person.

### Outcomes

The primary outcome was mental health recovery at 3 months assessed with the Recovery Assessment Scale-revised (RAS-r). This outcome assesses an individual’s orientation toward recovery and self-management across 5 domains: (1) personal confidence and hope, (2) willingness to ask for help, (3) goal and success orientation, (4) reliance on others, and (5) not dominated by symptoms [20]. The study intervention claims to promote self-management specifically, and the RAS-r was chosen as an outcome relevant to participants across all diagnoses. The use of the RAS-r also reflects the current *recovery era* for mental health policy and services with the focus of treatment shifting to consumers finding satisfying and fulfilling lives, rather than being symptom free [20]. The RAS-r is the most widely used and validated recovery assessment tool and has been studied in several patient populations and shown to correlate with symptoms and function [20]. It is a 24-item scale, with all items scored on a 5-point scale from *strongly disagree* to *strongly agree*, with total scores ranging from 24 to 120.

Secondary outcomes were the PHQ-9 to assess symptoms of depression, Generalized Anxiety Disorder Questionnaire-7 item (GAD-7) to assess symptoms of anxiety, EuroQOL 5-dimension quality of life questionnaire (EQ-5D-5L) to assess quality of life, and the Community Integration Questionnaire (CIQ) [21]. The PHQ-9 scale contains 9 items rated on a 4-point Likert scale from *never* to *almost every day*, with a higher score representing more symptoms [22]. The GAD-7 scale has 7 items rated on a 4-point Likert scale from *never* to *almost every day*, with a higher score representing a higher likelihood of an anxiety disorder. The EQ-5D-5L questionnaire from the EuroQOL group [23] comprises 5 dimensions: mobility, self-care, usual activities, pain or discomfort, and anxiety/depression, which are rated on 5 levels and can be interpreted as an index score by comparing with available normative values for adult populations. The EQ-5D-5L is paired with a visual analog scale (VAS) rated from 0 to 100 to assess perceived overall health at the time of survey completion. The CIQ consists of 15 items and is intended as a brief, reliable measure of a person’s level of integration into the home and community. The overall score can range from 0 to 29 with a higher score indicating better integration [24].

Actual BWW utilization data were obtained for all ITG participants including activation of account and number of logins.

### Sample Size

The target sample size for our trial was set by the sponsors at 1000 participants. We aimed to recruit this number of participants, with an expectation that loss to follow-up would be approximately 30% based on data from other similar trials [25]. We calculated the minimal detectable difference between the 2 treatment groups for a linear regression analysis controlling for baseline score assuming a 0·8 correlation between baseline and 3-month follow-up RAS-r measurements. Assuming 30% loss to follow-up, for a sample size of 700 after attrition, allocated in a 2:1 ratio, using an alpha of .05 and power of 0.9, the minimal detectable difference was 1.35 on the RAS-r.

### Randomization, Concealment, and Blinding

Participants were randomized 2:1 to the ITG and DTG groups. A 2:1 randomization ratio was used to offer the intervention immediately to a higher number of participants and increase recruitment. Randomization sequences, using block sizes of 3 or 6 were computer generated by an organization external to the research team, with stratification by site and recruitment setting. Group allocation sequence was concealed but once allocated, participants were not blinded. Participants allocated to the DTG group received a telephone or email notification that they would gain access to the website after a 3-month delay. Most follow-up data were collected by Web-based survey, but in the case where follow-up data were collected by an assessor, the assessor was not the same as the person who did the initial data collection, as this person also disclosed randomization to the participant and would not be blinded at follow-up. Data analysts remained blinded throughout the study.

### Statistical Analysis

The primary outcome, RAS-r, was analyzed at 3 months for all participants regardless of whether they activated their BWW account, but individuals with missing data at 3 months were excluded. We planned to repeat the analysis using a marginal structural model to account for attrition; however, owing to higher than expected attrition that was nonrandom, this analysis was not completed. RAS-r scores at 3 months were modelled with a linear regression controlling for baseline RAS-r score and treatment group. A second analysis controlled for prespecified covariates including baseline PHQ-9, baseline GAD-7, age, gender, recruitment setting, and age of first onset of mental health problems. The same analysis was repeated for all secondary outcomes at 3 months. Analyses were completed after data collection was complete. BWW utilization data were analyzed descriptively to illustrate uptake of the intervention in the ITG participants only.

### Exploratory Analysis

Among ITG participants who completed the 3-month outcome measures, we examined whether there was an association between actual utilization of the application and the primary and secondary outcomes. Utilization of the application was defined by number of BWW logins, with users categorized into 4 groups based on distribution quartiles: 0 to 1 login, 2 to 3 logins, 4 to 9 logins, and 10 or more logins. Owing to degree of missingness, primary and secondary outcomes were separately modeled with repeated measures ANOVA using time as a repeated measure. We tested for an interaction between BWW use and time, after adjusting for same covariates as in the main analysis. Significant main effects were explored with post-hoc Bonferroni tests adjusted for multiple comparisons.

## Results

**Figure 5:**
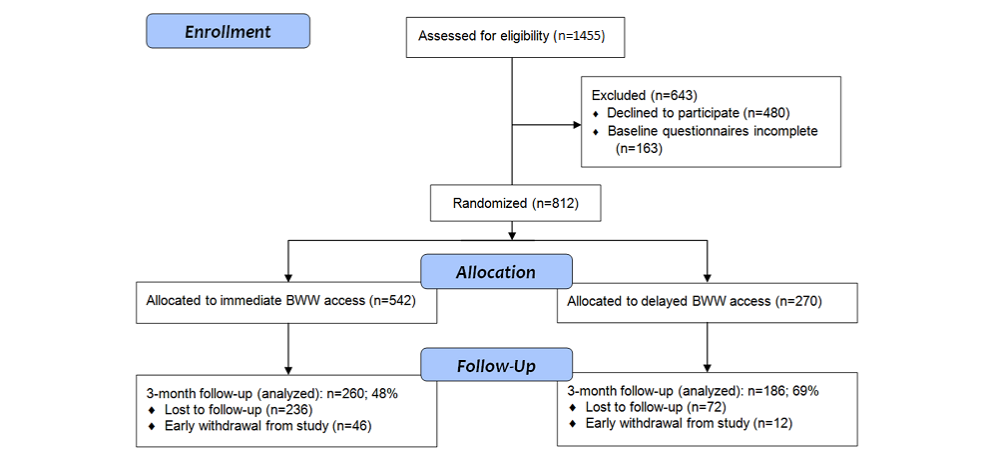
CONSORT flow diagram of participants through the trial. BWW: Big White Wall.

We approached 1455 individuals, of whom 975 (67.0%) consented. Of the 975, 163 (16.7%) did not complete the baseline assessment questionnaire, leaving 812 to be randomized, 270 (33.3%) to DTG and 542 (67.7%) to ITG (see Figure 5). Distribution of participants across recruitment settings was as follows: adult mood and anxiety psychiatry programs (n=294/812, 36.2%), youth mood and anxiety programs (n=42/812, 5%), mood and anxiety psychotherapy programs (n=73/812, 9%), emergency department/urgent care clinic (n=139/812, 17.1%), borderline personality/trauma therapy programs (n=114/812, 14.0%), and substance use program (n=150/812, 18.5%). Follow-up was achieved for 446/812 (54.9%) at 3 months, 260/542 (48.0%) in the ITG group and 186/270 (68.9%) in the DTG. At baseline, the randomized groups were well-balanced in terms of sociodemographic, mental health variables, and previous 3-month health care utilization (Table 1).

**Table 1:** Baseline characteristics of study participants by group.

Variables		Immediate Treatment Group (n=542)^a^	Delayed Treatment Group (n=270)^a^
Age (years), mean (SD)		41.5 (13.4)	40.0 (13.9)
**Gender, n (%)**			
	Male	143 (27)	59 (22)
	Female	391 (73)	207 (78)
	Transgendered	1 (0)	1 (0)
**Ethnicity, n (%)**			
	White	440 (82)	219 (83)
	Non-white	98 (17)	46 (18)
**Relationship status, n (%)**			
	In a relationship	286 (53)	127 (47)
	Not in a relationship	252 (47)	142 (52)
**Employment status, n (%)**			
	Full-time (including homemaker with young children)	181 (33)	96 (35)
	Part-time/volunteer/homemaker without young children	100 (19)	41 (15)
	Not working (retired owing to age or actively looking for work)	33 (13)	26 (14)
	Not working (not looking for work)	84 (35)	57 (31)
**Household income in Can $, n (%)**			
	<35K	238 (48)	126 (52)
	35K-50K	56 (11)	38 (16)
	50K-80K	82 (16)	25 (10)
	>80K	123 (25)	52 (22)
Age first experienced mental health problems (years), mean (SD)		18.7 (12.5)	19.0 (12.7)
Age first sought help (years), mean (SD)		26.7 (12.9)	26.3 (13.0)
Taking medication at baseline, n (%)		438 (81)	204 (76)
Previous 3-month hospitalization, n (%)		47 (9)	23 (9)
Previous 3-month emergency room visit, n (%)		77 (14)	27 (10)
**Agree with: Self-help tools helpful for people with mental health problems, n (%)**			
	Definitely agree	203 (38)	110 (41)
	Somewhat agree	310 (58)	151 (56)
	Somewhat or completely disagree	24 (4)	8 (3)
**How much expected improvement in mental health through BWW^b,c^, n (%)**			
	Less than 50%	194 (38)	110 (42)
	50%	141 (26)	76 (29)
	More than 50%	179 (35)	76 (29)

^a^Percentages calculated after missing data removed.

^b^Responses were recorded in 10% increments but based on their distribution have been recategorized.

^c^BWW: Big White Wall.

There were some differences between those who were lost to follow-up and those who completed the 3-month survey overall and in each group (see Multimedia Appendices 1 and 2). Overall, the survey completers were older, and more likely to be working full-time or unemployed and not looking for work. ITG survey completers were more likely to activate their BWW accounts and have more logins than noncompleters in that group, with the same age and employment patterns as overall. In the DTG group, the only significant difference found was for recruitment setting; those from youth and emergency or urgent care settings were most likely to complete follow-up. A proportion of survey noncompleters from both the ITG and DTG groups withdrew early from the study (46 out of 282 ITG survey noncompleters (16%) and 12 out of 84 (14%) DTG survey noncompleters).

### Primary Outcome

The primary analysis showed a statistically significant difference between the ITG and DTG groups for the RAS-r at 3 months, with the ITG participants having an RAS-r score on average 5.34 points higher than the DTG participants at 3 months (Table 2). In the adjusted model, the effect remained significant but slightly lower in magnitude (Table 2).

**Table 2:** Baseline and 3-month primary and secondary outcomes among survey completers with linear regression results for outcomes at 3 months.

Outcome		Immediate treatment group (n=260), mean (SD)		Delayed treatment group (n=186), mean (SD)		Unadjusted treatment effect size (95% CI)^a^	Adjusted treatment effect size (95% CI)^a,b^
		Baseline	3 months	Baseline	3 months		
**Primary outcome**							
	Recovery Assessment Scale, revised	77.4 (14.0)	83.3 (15.1)	76.3 (14.1)	77.2 (14.6)	5.32 (3.33 to 7.31)	4.97 (2.90 to 7.05)
**Secondary outcomes**							
	Patient Health Questionnaire-9 item	14.8 (6.9)	11.5 (6.4)	16.0 (6.5)	14.2 (6.8)	−1.95 (−2.94 to −0.95)	−1.83 (−2·85 to −0·82)
	Generalized Anxiety Disorder Questionnaire-7 item	11·5 (5·6)	9·1 (5·3)	12·3 (5·6)	11·4 (5·7)	−1.75 (−2.60 to −0.90)	−1.56 (−2.42 to −0.70)
	EuroQOL 5-dimension quality of life questionnaire	0.68 (0.16)	0.71 (0.17)	0.68 (0.16)	0.69 (0.15)	0.014 (**−**0.009 to 0.036)	0.008 (**−**0.015 to 0.031)
	EuroQOL Visual Analog Scale	56.8 (19.2)	58.8 (21.5)	55.1 (19.8)	55.4 (21.9)	2.55 (**−**1.17 to 6.26)	1.68 (**−**1.97 to 5.32)
	Community Integration Questionnaire	16.9 (5.0)	17.0 (5.2)	16.7 (4.6)	17.1 (4.8)	−0.32 (**−**0.93 to 0.29)	−0.30 (**−**0.93 to 0.33)

^a^Delayed treatment group is the reference group in all analyses; all models include baseline score.

^b^Adjusted for age, sex, recruitment setting, baseline PHQ-9 score, baseline GAD-7 score, and age of first onset of mental health problems.

### Secondary Outcomes

PHQ-9 and GAD-7 scores were significantly lower in the ITG group compared with the DTG group in the main and adjusted analyses (Table 2). No statistically significant differences were found for EQ-5D-5L index score, EQ-VAS, or CIQ (Table 2).

### Participant Safety

The data safety monitoring board reviewed data at 3 and 6 months after the start of recruitment and did not find any substantial increase in suicidal ideation between groups to warrant investigation or early termination. Over the course of the study, one death was reported to the study team and deemed unrelated to the intervention.

### Uptake, Use, and Satisfaction With the Intervention

#### Big White Wall Activation

Among the 542 participants who were randomized to receive immediate access to the BWW, 76 (14%) never activated their BWW account during the study period. Half of these individuals (n=39, 51%) withdrew early from the study. Reasons for early withdrawal included the following: technical issues, loss of perceived need or interest, and lack of time owing to competing priorities.

#### Utilization

There was large variability in the range of total number of times that participants logged on to the site, from 0 to 236 times. The mean number of logins was 8.7, with a standard deviation of 18.1, a median of 2, and a mode of 1. Only 58% (312/542) of participants logged on 2 or more times, with approximately 20% of all participants accounting for 80% of the total logins.

#### Exploratory Analysis

There was no significant interaction between BWW use and time for the primary outcome of RAS-r. The interaction was significant for PHQ-9 (*F*_3,257_=4.14; *P*=.007) and GAD-7 (*F*_3,267_=3.89; *P*=.009). In post-hoc analysis for PHQ-9, a significantly greater reduction in score over time was present for the groups with 10 or more logins and 2 to 3 logins, relative to the 0 to 1 login group (4.14 points vs 1.43, *P*=.03 and 5.00 vs 1.43, *P*=.02, respectively). In post-hoc analysis for GAD-7, only the group with 2 to 3 logins had a significantly greater reduction in score over time compared with the group with 0 to 1 login (4.36 points vs .91 points, *P*=.006). There was no significant effect of the interaction between BWW use and time on the other secondary outcomes.

## Discussion

### Principal Findings

Immediate access to the BWW resulted in small, significant improvements in mental health recovery, as well as depressive and anxiety symptoms at 3 months compared with those randomized to delayed access. These statically significant findings are limited by high, differential drop out between treatment groups and overall, and were below minimal clinically important differences for these outcome measures (eg, a difference of 1.8 on the PHQ-9 where a clinically important change is 5 points) [22]. Engagement with the intervention varied highly and interestingly, we observed the commonly found Pareto principle for population effects—also known as the *80/20* rule [26]. That is, 20% of users accounted for approximately 80% of the activity. We found some evidence of a user effect whereby those who completed follow-up and engaged with the application were more likely to experience improvements in symptoms of depression and anxiety. This was not linear, however, with the user group having 2 to 3 logins experiencing the most consistent improvements relative to the lowest user group. To our knowledge, this is the first randomized evaluation of the BWW, and one of a few large multicenter pragmatic trials of any multicomponent internet-based mental health intervention. The study population represents more treatment-refractory and severely symptomatic individuals than are usually included in studies of Web-based mental health applications.

### Comparison With Other Studies

Although the BWW is unique in comparison with other studied applications, the observed low engagement is similar [25]. A comparatively large pragmatic trial that examined 2 computerized modular CBT programs with added telephone support reported that fewer than 20% of participants had completed all modules at follow-up [25]. Modular-style courses are only one component of the BWW, the other components being peer support, artistic self-expression, and more general psychoeducation. Compared with a modular program, it is more difficult to define an adequate *dose* for multicomponent interventions such as the BWW [13]. We examined different self-directed *doses* of the BWW based on logins and did not find a linear relationship between higher use and better outcomes, although it appeared that some engagement may have been better than none. Some users may benefit from a few targeted uses to get direction or motivation. A systematic review examining the relationship between e-therapy adherence and outcomes reported that studies targeting depression did not find a significant impact of total number of logins on outcomes in contrast to studies examining physical health outcomes like weight management or smoking [9]. Studies using modular interventions, however, did report that module completion led to better outcomes. Conversely, one of the main features of the BWW used more often by the higher engagers is its moderated virtual community of peers [15], an intervention for which the evidence has yielded some very mixed results [27,28]. Some authors have described a potential harm through aggravated symptoms and what Takahashi et al termed the *downward depressive spiral* which was linked to higher depressive tendencies at baseline [29]. This may have tempered the improvements in the higher engager groups or conversely may have led to early disengagement from the application as a result of negative reactions to the peer community.

### Limitations

This study experienced loss to follow-up that was proportionally higher in the intervention group. High drop out of up to 50% in trials of Web-based mental health interventions has been reported [10] and retention in digitized trials has specifically been discussed as a challenge [30]. In our study, the issues encountered with our automated survey were addressed quickly but may have impacted survey completion. More specifically, other studies of Web-based mental health programs have also reported disproportionate dropout in the intervention group [10]. This trend likely represents some study disengagement for intervention-specific reasons such as dislike of the intervention format or lack of perceived utility. In this study, some very early technology issues with BWW activation likely affected early user engagement and the Web-based platform did not include a mobile version of the site or application which could have deterred use for some. To partially address this limitation, we conducted the exploratory analysis on study completers.

We chose the pan-diagnostic RAS-r as the primary outcome, given that our study sample was recruited from a range of mental health settings and participants had a range of mental health needs. The promise of internet-based mental health interventions specifically to support recovery has been discussed [31]; however, this outcome lacks established clinically important cutoffs. Secondary outcomes may have been affected by the lack of diagnostic specificity relative to other studies that have focused on specific diagnoses such as depression and anxiety [6]. In this study, we evaluated the BWW as a solution for any mental health or addiction-related need; although we adjusted for recruitment program, it is possible that the BWW may work better for certain diagnostic or need subgroups, which represents an area for further evaluation.

### Conclusions and Policy Implications

From this trial, we cannot definitively conclude the effectiveness of this or similar solutions at a population level for individuals accessing specialized mental health care. However, internet-based interventions built on evidence-based principles of mental health care are likely to be beneficial where users are motivated to engage and where the format of the application is a good fit. Determining this subset and/or how to effectively motivate more people to engage with the interventions is a critical next step. Pragmatic studies that build in process evaluations and subset analyses that examine moderators of use and related outcomes are required. This study was undertaken as part of a large provincial implementation program with multiple research, clinical, and policy stakeholders involved throughout, a type of integrated, real-world approach to evaluation that is essential to establish effective health systems solutions [32].

## Appendix

Multimedia Appendix 1 Baseline characteristics of study participants who did and didn’t complete the follow-up measures at 3 months.

Multimedia Appendix 2 Baseline characteristics of study participants by group, who did and didn’t complete the follow-up measures at 3 months.

Multimedia Appendix 3 CONSORT-eHEALTH checklist (V 1.6.1).

## Abbreviations

BWW: Big White Wall
CIQ: Community Integration Questionnaire
DTG: delayed treatment group
EQ-5D-5L: EuroQOL 5-dimension quality of life questionnaire
GAD-7: Generalized Anxiety Disorder Questionnaire-7 item
ITG: immediate treatment group
PHQ-9: Patient Health Questionnaire-9 item
RAS-r: Recovery Assessment Scale, revised
VAS: visual analog scale
