# Supplementary material for: A Web-Based Mental Health Platform for Individuals Seeking Specialized Mental Health Care Services: Multicenter Pragmatic Randomized Controlled Trial
Source: J Med Internet Res. 2019 Jun 4;21(6):e10838. doi: 10.2196/10838 (PMC6684216; doi:10.2196/10838)
Supplement: Multimedia Appendix 1 [file jmir_v21i6e10838_app1.pdf]

Variable	Follow-up complete n = 448	Lost to follow-up n = 364	*P*-value
**Recruitment Setting, No. (%)**			
Adult Mood and Anxiety Psychiatry Programs	169 (57)	125 (43)	.83
Youth Mood and Anxiety Programs	24 (57)	18 (43)	
Adult Mood and Anxiety Psychotherapy Program	36 (49)	37 (51)	
Emergency Department/Urgent Care	77 (55)	62 (45)	
Borderline Personality Disorder/Trauma Therapy Programs	63 (55)	51 (45)	
Substance Use Program	79 (53)	71 (47)	
**Age, mean (SD)**	41.5 (13.8)	39.4 (13.2)	**.04**
**Gender, No. (%)**			
Male	107 (53)	95 (47)	.87
Female	336 (56)	262 (44)	
Transgendered or not specified	5 (56)	4 (44)	
**Ethnicity, No. (%)**			
White	372 (56)	287 (44)	.13
Non-white	76 (50)	77 (50)	
**Relationship Status, No. (%)**			
In a relationship	240 (58)	175 (42)	.12
Not in a relationship	208 (52)	189 (48)	
**Employment Status, No. (%)**			
Full-time (including. homemaker with young children)	165 (60)	112 (40)	**.03**
Part-time/volunteer/homemaker without young children	67 (48)	74 (52)	
Not working - retired due to age or actively looking for work	57 (48)	61 (52)	
Not working – not looking for work	159 (59)	111 (41)	
**Household income in $CAD, No. (%)**			
<$35K	165 (52)	150 (48)	.36
$35K - $50K	57 (61)	37 (39)	
$50K - $80K	65 (60)	43 (40)	
>$80K	98 (56)	78 (44)	
**Age first experienced mental health problems, mean (SD)**	18.8 (12.5)	18.8 (12.5)	.94
**Age first sought help, mean (SD)**	26.3 (12.7)	26.8 (13.2)	.57
**Taking medication at baseline, No. (%)**	359 (56)	283 (44)	.70
**Agree with: Self-help tools helpful for people with mental health problems, No. (%)**			
Somewhat or definitely agree	431 (55)	349 (45)	.81
Somewhat or completely disagree	17 (53)	15 (47)	
**How much expected improvement in mental health through BWW[a], No. (%)**			
Less than 50%	178 (58)	129 (42)	.41
50%	115 (52)	105 (48)	
More than 50%	155 (54)	130 (46)	
**Did not activate BWW account, No. (%) (ITG only)**	16 (17)	77 (83)	**<.001**
**Total BWW logins, mean (SD) (ITG only)[b]**	12.9 (22.1)	5.4 (12.8)	**<.001**

[a]Responses were recorded in 10% increments but based on their distribution, have been recategorized.
[b]Inactivated accounts counted as 0 logins.
Percentages calculated after missing data removed.
ITG – Immediate Treatment Group
