# Supplementary material for: A Web-Based Mental Health Platform for Individuals Seeking Specialized Mental Health Care Services: Multicenter Pragmatic Randomized Controlled Trial
Source: J Med Internet Res. 2019 Jun 4;21(6):e10838. doi: 10.2196/10838 (PMC6684216; doi:10.2196/10838)
Supplement: Multimedia Appendix 2 [file jmir_v21i5e10838_app2.pdf]

Variable	ITG Follow-up complete n=262	ITG Not complete n=280	*P*-value	DTG Follow-up complete n=186	DTG Not complete n=84	*P*-value
**Recruitment Setting**						
Adult Mood and Anxiety Psychiatry Programs	101 (51)	96 (49)	.33	68 (70)	29 (30)	**.04**
Youth Mood and Anxiety Programs	12 (44)	15 (56)		12 (80)	3 (20)	
Adult Mood and Anxiety Psychotherapy Program	22 (45)	27 (55)		14 (58)	10 (42)	
Emergency Department/Urgent Care	38 (41)	54 (59)		39 (83)	8 (17)	
Borderline Personality Disorder/Trauma Therapy Programs	44 (57)	33 (43)		19 (51)	18 (49)	
Substance Use Program	45 (45)	55 (55)		34 (68)	16 (32)	
**Age, mean (SD)**	42.6 (13.4)	39.6 (13.2)	**.008**	39.8 (14.2)	38.9 (13.3)	.62
**Gender, No. (%)**						
Male	69 (48)	75 (52)	.98	38 (64)	21 (36)	.77
Female	191 (49)	202 (51)		145 (70)	62 (30)	
Transgendered or not specified	2 (40)	3 (60)		3 (75)	1 (25)	
**Ethnicity, No. (%)**						
White	222 (50)	222 (50)	.10	152 (69)	69 (31)	.93
Non-white	40 (41)	58 (59)		34 (69)	15 (31)	
**Relationship Status, No. (%)**						
In a relationship	147 (51)	141 (49)	.18	93 (65)	50 (35)	.15
Not in a relationship	115 (45)	139 (55)		93 (73)	34 (27)	
**Employment Status, No. (%)**						
Full-time (including. homemaker with young children)	96 (53)	85 (47)	**.04**	69 (72)	27 (28)	.73
Part-time/volunteer/homemaker without young children	41 (41)	59 (59)		26 (63)	15 (36)	
Not working - retired due to age or actively looking for work	29 (38)	47 (62)		28 (67)	14 (33)	
Not working – not looking for work	96 (53)	86 (47)		63 (72)	25 (28)	
**Household income in $CAD, No. (%)**						
<$35K	97 (47)	111 (53)	.18	68 (64)	39 (36)	.06
$35K - $50K	25 (45)	38 (55)		32 (84)	6 (16)	
$50K - $80K	45 (54)	31 (46)		20 (80)	5 (20)	
>$80K	64 (52)	60 (48)		34 (65)	18 (35)	
**Age first experienced mental health problems, mean (SD)**	18.8 (12.4)	18.6 (12.5)	.79	18.6 (12.7)	19.7 (12.6)	.53
**Age first sought help, mean (SD)**	26.8 (12.9)	26.6 (12.9)	.91	25.7 (12.5)	27.5 (14.0)	.29

**Taking medication at baseline, No. (%)**	213 (49)	225 (51)	.98	146 (72)	58 (28)	.22
**Agree with: Self-help tools helpful for people with mental health problems, No. (%)**						
Somewhat or definitely agree	250 (48)	268 (52)	.87	181 (69)	81 (31)	.71
Somewhat or completely disagree	12 (50)	12 (50)		5 (63)	3 (37)	
**How much expected improvement in mental health through BWW[a], No. (%)**						
Less than 50%	96 (49)	99 (51)	.94	82 (73)	30 (27)	.12
50%	69 (48)	74 (52)		46 (60)	21 (40)	
More than 50%	97 (48)	107 (52)		58 (72)	23 (28)	

[a]Responses were recorded in 10% increments but based on their distribution, have been recategorized.
[b]Inactivated accounts counted as 0 logins.
Percentages calculated after missing data removed.
ITG – Immediate Treatment Group; DTG – Delayed Treatment Group
